# Crystal structure of bacterial haem importer complex in the inward-facing conformation

**DOI:** 10.1038/ncomms13411

**Published:** 2016-11-10

**Authors:** Youichi Naoe, Nozomi Nakamura, Akihiro Doi, Mia Sawabe, Hiro Nakamura, Yoshitsugu Shiro, Hiroshi Sugimoto

**Affiliations:** 1 Biometal Science Laboratory, RIKEN SPring-8 Center, 1-1-1 Kouto, Sayo, Hyogo 679-5148, Japan; 2 Department of Life Science, University of Hyogo, 3-2-1 Kouto, Kamigohri, Akoh, Hyogo 678-1297, Japan

## Abstract

Pathogenic bacteria remove iron from the haem of host tissues and use it as a catalytic center of many enzymes. Haem uptake by pathogenic bacteria is facilitated by the membrane-integrated haem importer, which belongs to the type II ATP-binding cassette (ABC) transporter. Here we present crystal structures of *Burkholderia cenocepacia* haem importer BhuUV complexed with the periplasmic haem-binding protein BhuT and in the absence of BhuT. The transmembrane helices of these structures show an inward-facing conformation, in which the cytoplasmic gate of the haem translocation pathway is completely open. Since this conformation is found in both the haem- and nucleotide-free form, the structure of BhuUV-T provides the post-translocation state and the missing piece in the transport cycle of the type II importer. Structural comparison with the outward-facing conformation reported for the haem importer ortholog HmuUV from *Yersenia pestis* gives mechanistic insights into conformational transitions and haem secretion during the haem transport cycle.

## Abstract (web summary)

Pathogenic bacteria acquire iron from heme cofactors imported by ABC heme transporters. Here the authors present crystal structures of *Burkholderia cenocepacia* heme importer BhuUV with and without the heme-binding protein BhuT, gathering mechanistic insight into the catalytic cycle of heme import.

## Graphical abstract

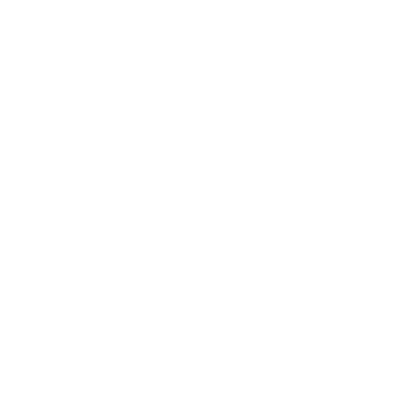

Iron is an essential element for almost all organisms, including animals, plants and microorganisms, because it forms the active center of iron-containing proteins involved in biosynthesis and the metabolism of many essential bioactive compounds. Some bacteria infecting or residing in higher organisms acquire iron from the haem (iron–porphyrin complex) of host haem-containing proteins, in particular haemoglobin in red blood cells. Most pathogenic Gram-negative bacteria transport haem across the plasma membrane using an ATP-dependent active transport system, the ATP-binding cassette (ABC) transporter system^1^,^2^. In this system, the haem is captured and shuttled by periplasmic haem-binding protein (PBP), and then imported into the cytoplasm through the transmembrane proteins for permease (TMD) and a nucleotide-binding ATPase subunit (NBD). The imported haem is degraded by the cytoplasmic protein haem oxygenase and the iron is extracted. The haem degradation reaction by haem oxygenase has been well characterized at the atomic level, but less is known about the haem import reaction across the cellular membrane, which is coupled with ATP hydrolysis by the ABC haem importer.

Woo *et al*.^3^ recently reported the crystal structure of the haem importer HmuUV from the pathogen *Yersinia pestis*. This is the first molecular structure of an ABC haem importer. The HmuUV structure in a nucleotide-free form consisted of the dimerized TMD (HmuU) and dimerized NBD (HmuV). This structure showed that the haem translocation pathway through HmuU is open toward the periplasmic side and is closed facing the cytoplasmic side, suggesting that HmuUV in this state is in an outward-facing conformation. The structure of HmuUV showed a fold similar to that of type II ABC importers such as the vitamin B_12_ transporter BtuCD-F system from *Escherichia coli* and the molybdate transporter MolAB (formerly HI1470/1) from *Haemophilus influenza*^4^.

The BtuCD-F system consisting of BtuC (TMD), BtuD (NBD) and BtuF (PBP) is a bacterial type II ABC importer, and has been structurally and functionally well characterized. The crystal structures of nucleotide-free BtuCD, adenylyl imidodiphosphate (AMP–PNP)-bound BtuCD, nucleotide-free BtuCD-F and AMP–PNP-bound BtuCD-F have been solved^5^,^6^,^7^,^8^. Crystallographic studies revealed that the structure of BtuCD is in the outward-facing conformation, irrespective of the presence or absence of the nucleotide analogue (AMP–PNP), and that of the BtuCD-F complex is in an asymmetric and occluded conformation in the nucleotide-free form. These structures of BtuCD-F in the four key states have revealed conformational rearrangement of the protein by nucleotide- and PBP-binding, leading to the proposed mechanism of structural change for vitamin B_12_ transport (Supplementary Fig. 1a).

Structures of the ABC haem importers in several states are needed for understanding the molecular mechanism of haem acquisition at the atomic level. Here we describe the structures of the type II haem importer system (BhuUV-T) from the Gram-negative pathogen *Burkholderia cenocepacia*. BhuUV-T is a close orthologue of HmuUV-T^9^,^10^, which is a complex of BhuU (TMD), BhuV (NBD) and BhuT (PBP). We determined the structures of not only the BhuUV complex, but also the complete BhuUV-T complex. These complexes exhibit the inward-facing conformation, which is different from that of HmuUV reported previously. In combination with previous HmuUV studies, this structural study provides mechanistic insights into the conformational transitions of the bacterial haem importer during its reaction cycle.

## Results

### Overall structure of haem transporter

**Figure 1:**
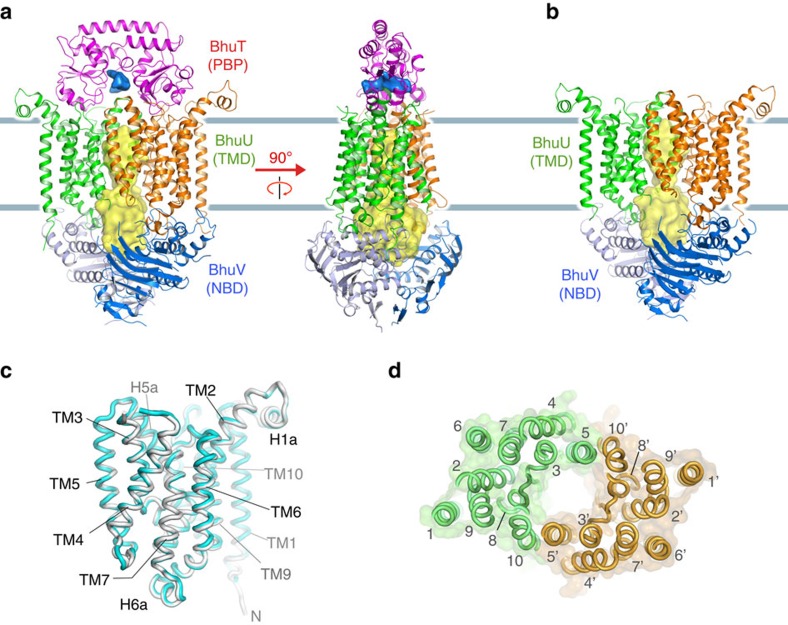
Crystal structures of the type II ABC haem transporters BhuUV-T and BhuUV. (**a**) Overall structure of the nucleotide-free form of *B. cenocepacia* haem transporter BhuUV complexed with BhuT. Each subunit is shown in a different colour. The large cavity of BhuUV open to the cytoplasm is represented by a yellow surface. The small cavity of BhuT in the haem-binding site open to the periplasm is shown as a blue surface. In the right panel, BhuUV-T was rotated by 90° about the vertical axis. (**b**) Overall structure of nucleotide-free BhuUV. (**c**) Superposition of the BhuU monomer of BhuUV-T (grey) and BhuUV (cyan). (**d**) Arrangement of the transmembrane helices of BhuU dimer viewed from periplasmic side along the two-fold dimer axis.

Recombinant BhuUV and BhuT were separately overexpressed in *E. coli* and purified. The BhuUV-T complex was co-purified using affinity and size-exclusion chromatography. The crystal structures of BhuUV-T and BhuUV were determined in the nucleotide-free form at 3.2 and 2.8 Å resolution, respectively (Fig. 1a,b). The structure of BhuUV consists of two TMDs (BhuU) and two NBDs (BhuV), while BhuUV-T is a heteropentamer complex comprising two TMDs, two NBDs and one PBP (BhuT). The BhuU monomers in BhuUV-T and BhuUV also shows very similar main chain conformation with root mean-squared deviation (r.m.s.d.) of 0.7 Å (Fig. 1c). Both structures showed clear electron density for the BhuU and BhuV subunits (Supplementary Fig. 2), enabling us to build 94% of the total residues in the final model. Each subunit showed a conserved fold, and the overall architectures of the BhuUV-T and BhuUV structures were similar to those of other type II ABC transporters^3^,^4^,^8^.

The BhuU dimer contains 20 transmembrane (TM) helices and is fairly symmetric, and its two polypeptide chains (monomers) have similar conformations, with an r.m.s.d. of 0.6 Å over 308 (85%) Cα atoms. As illustrated in Fig. 1d, the BhuU monomers dimerize through hydrophobic interactions between TM5 and TM10 of each monomer, and the haem-translocating channel (∼50 Å long) is created at this interface along the dimer axis (Fig. 1a,b). The channel is extended from the BhuT–BhuU interface to the BhuV–BhuV interface. The region between TM6 and TM7 of the cytoplasmic side of BhuU contains a coupling helix (Supplementary Fig. 3) that extensively interacts with BhuV and plays an important role in the transmission of the conformational change of NBD to TMD in response to ATPase activity^11^,^12^. ATP binds to BhuV in the cytoplasm to be hydrolysed and utilized for the conformational change in the haem-transport reaction. The conserved motifs for ATP recognition by BhuV, namely, the P-loop (Walker A), LSGGQ motif (ABC signature motif) and Q-loop (Supplementary Figs 2c and 3a), show an arrangement similar to that observed in the NBD of other ABC transporters. All these structures are key common features observed in the TMD and NBD of type II importers^4^,^8^.

### Inward-facing conformation of BhuUV-T and BhuUV

**Figure 2:**
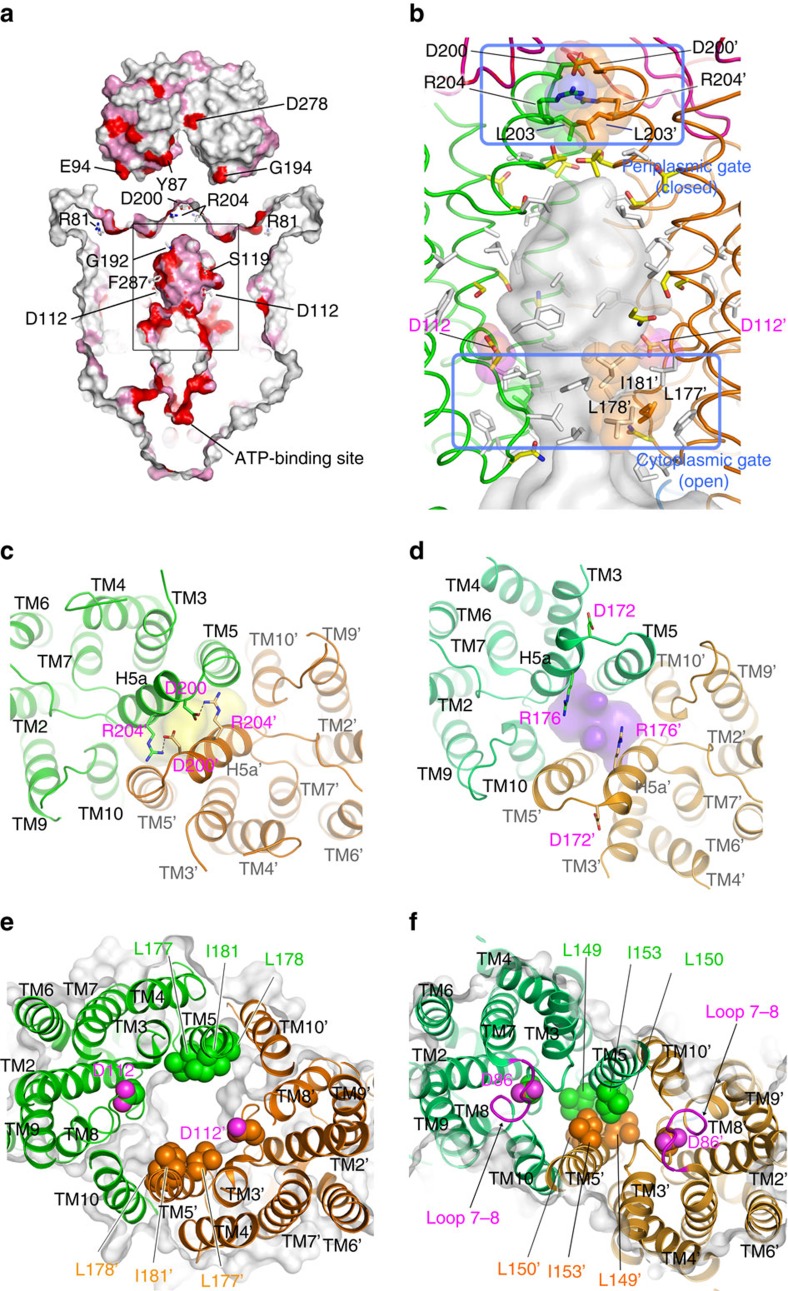
Residues aligned in the channel of haem transporter. (**a**) Surface representation of conserved (red) and conservatively substituted residues (pink) of BhuUV-T among the functionally characterized haem transporters. BhuUV and BhuT are separated for clarity and the conserved residues are labelled. (**b**) Translocation channel of BhuUV-T created by hydrophobic (white sticks) and polar residues (yellow sticks). The periplasmic gate is closed by the interaction between three residues (D200, L203 and R204) of helices H5a. The cytoplasmic gate formed by hydrophobic residues around L177 is open. (**c**) Periplasmic gate of BhuUV-T viewed from the periplasmic side along the two-fold axis. The gate is completely sealed by interactions of the two H5a helices with the salt bridge between D200 and R204. The long channel at the bottom of H5a and extending to the cytoplasm is shown as a yellow surface. (**d**) Periplasmic gate of HmuUV. The two H5a helices are separated from each other and the cavity (purple surface) is open to the periplasmic side. (**e**) Cytoplasmic gate of BhuUV-T viewed from the periplasmic side. Hydrophobic residues L177, L178 and I181 of TM5 are separated from those of the other monomer to open the cytoplasmic gate. D112 is the only charged residue in the BhuU dimer channel. (**f**) The cytoplasmic gate of HmuU is sealed due to hydrophobic interactions involving L149, L150 and I153 of TM5. D86 corresponds to D112 of BhuU and is buried by the loop residues between TM7 and TM8 (magenta).

**Figure 3:**
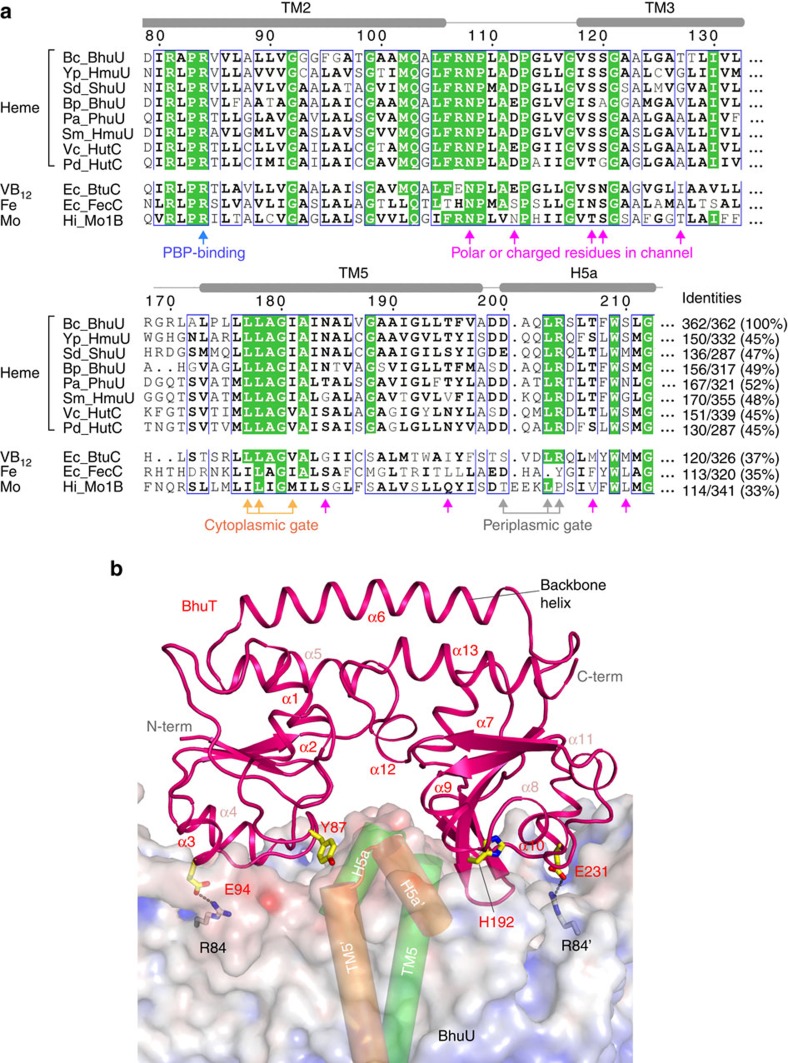
Sequence conservation of the residues in the channel and PBP-TMD interface. (**a**) Sequence alignment of regions of TM2-TM3 and TM5-H5a in the haem importers, vitamin B_12_ transporter, iron-chelate transporter and molybdate transporter. Bc, *Burkholderia cenocepacia*; Yp, *Yersinia pestis*; Ec, *Escherichia. coli*; Sd, *Shigella dysenteriae*; Bp, *Bordetella pertussis*; Pa, *Pseudomonas aeruginosa*; Sm, *Sinorhizobium meliloti*; Vc, *Vibrio cholerae*; and Pd, *Photobacterium damselae*; Hi, *Haemophilus influenza*. GenBank accession numbers are listed in Online Methods. (**b**) Interface between BhuT (magenta ribbon) and dimeric BhuU (surface representation) in the crystal structure of the BhuUV-T complex. The N- and C-terminal domains of BhuT are bound in the groove above the BhuU dimer. The haem-binding pocket of BhuT is partially occupied by the two H5a helices of the BhuU dimer. The conserved residue pairs (R84–E94 and R84–E231) for salt bridge formation at the interface are shown as stick models. Y87 of BhuT is the ligand for haem.

Figure 2a,b shows the haem-translocating channel of BhuUV-T. The periplasmic side of the channel is blocked by interactions between a pair of H5a helices: this blockage involves a pair of salt bridges between D200 of one monomer and R204 of the other (Fig. 2b,c), and hydrophobic interactions between the L203 side chains of each monomer (Fig. 2b). These three residues are highly conserved in bacterial haem importers^3^ (Fig. 3a). The interactions between D172 and R176 in the corresponding region of HmuUV^3^ (Fig. 2d) are lost and thus these helices form the periplasmic gate for the haem-transporting channel. The periplasmic gate is completely closed in our BhuUV and BhuUV-T structures (Fig. 2a,c), but open in the HmuUV structure (Fig. 2d and Supplementary Fig. 3b).

In contrast, the cytoplasmic gate is observed at the bottom of TM5 in the HmuUV structure (Fig. 2f). The cytoplasmic gate in HmuUV is closed due to hydrophobic interactions involving L149, L150 and I153. The corresponding region of BhuUV and BhuUV-T is located in the middle of the haem-transporting channel. L177, L178 and I181 create a hydrophobic patch and the cytoplasmic gate is open, as shown in Fig. 2b,e. The diameter of the gate is 13 Å (L177-L177 distance) and is the narrowest part of the channel, but the gate is wide enough for the haem (∼10 Å) to pass through. L177, L178 and I181 are highly conserved in the haem importers (Fig. 3a). The structural characteristics of the channel, in which the periplasmic gate is closed and the cytoplasmic gate is open, allowed assignment of the observed structures of BhuUV-T and BhuUV to the inward-facing conformation. This is in sharp contrast to the outward-facing conformation previously observed in HmuUV, where the periplasmic gate is open and the cytoplasmic gate is closed^3^.

The channel for haem translocation (import) extends from the region below H5a to the cytoplasmic solvent region via the BhuU–BhuU and BhuV–BhuV dimerization interface (Fig. 2a,b). The wall of this channel is formed by TM3, 5, 8 and 10; in total, 28 hydrophobic residues, 9 polar residues (N108, S119, S120, T127, N184, T195, T207, S210 and T319) and only 1 charged residue (D112) are found per BhuU monomer (Fig. 2b,e). Most of the residues in the BhuU channel are conserved or conservatively substituted among the haem transporters (Fig. 2a). No potential ligand for the haem iron, such as the His, Cys, Tyr or Met found in hemoproteins, is found in the translocating channel, suggesting that there is no stable binding/trapping site for haem in the translocation pathway. Instead, hydrophobic residues might transiently interact with and bind haem during translocation. This is in sharp contrast with the *E. coli* maltose transporter MalFGK_2_ (refs 13, 14), which has a hydrophilic binding site to recognize the substrate maltose specifically within the translocation channel.

### Interaction of BhuT with BhuU in BhuUV-T

In the BhuUV-T crystal structure, the BhuUV subunits show well-defined electron density, whereas BhuT exhibits poor electron density (Supplementary Fig. 2a) and high temperature factors, particularly in the loops regions. Poorly defined density has also been reported for the periplasmic protein BtuF in the BtuCD-F complex^7^ and is caused by the presence of two orientations of BtuF with respect to the BtuCD in the crystal^15^. A similar explanation may hold for BhuT in the BhuUV-T crystal: the alternative orientation of BhuT related by a 180° rotation along the dimer axis of BhuU–BhuU might be allowed, because BhuT is free from crystal packing contact. However, only one orientation could be built in the final model (see the Methods section).

BhuT consists of two topologically similar domains—the N- and C-terminal domains—and each comprises a β-sheet surrounded by five or six short α-helices (Fig. 3b). The two domains are connected by a single α-helix (backbone helix) and these two connected domains are characteristic of the class III PBPs. The structure of the BhuUV-T complex revealed that BhuT interacts with the periplasmic surface of BhuU. In this interaction, the α3–α4 helices of the N-terminal domain and the α9–α10 helices and a portion of the β-strands of the C-terminal domain of BhuT are bound into a shallow pocket on the periplasmic surface of the BhuU dimer. This binding is mediated by van der Waals contacts, as well as salt bridges between E94 or E231 of BhuT with R84 of BhuU. E94, E231 and R84 are highly conserved in the type II ABC importers and may play important roles in the formation of the complex^3^,^16^. As expected from the structures of the isolated PBPs^17^, a cleft between the two domains of BhuT contains several hydrophilic residues (Y87, R89, Q88, H192 and Y225) and provides a haem-binding site. Most of the space in the cleft of the BhuUV-T complex is occupied by the two H5a helices of the BhuU dimer, and only a very small space is left between BhuT and BhuUV. The structure suggests that the release of haem from the BhuT cleft is facilitated on formation of the BhuUV-T complex and that haem cannot bind into this cleft from the outside of the complex.

The functional effect of BhuT binding to BhuUV was examined by measuring the ATP hydrolysis activities of BhuUV in the absence and presence of BhuT. BhuUV dissolved in detergent solution had a *K*_m_ of 28 μM and a *V*_max_ of 5.0 μmol min^−1^ mg^−1^ (Supplementary Fig. 4) in the absence of PBP (BhuT). The addition of haem-free BhuT resulted in a 1.4-fold increase in the *V*_max_ of BhuUV and *K*_m_ also increased (*K*_m_ of 92 μM, *V*_max_ of 7.1 μmol min^−1^ mg^−1^). A similar level of basal ATPase activity and stimulation by PBP is reported for other type II importers^3^,^18^, whereas different ATPase stimulation is reported for type I importers (maltose transporter MalFGK_2_ and His permease)^19^,^20^. For example, in the maltose transporter, basal activity is observed in detergent solution but not in a reconstituted liposome system. Our results revealed that BhuUV in a reconstituted system also shows a basal ATPase activity and a small effect of BhuT (1.4-fold increase in activity; Supplementary Fig. 4).

Inspection of the superposition of the two structures revealed that the dissociation of BhuT shows a slight change in the relative orientation of BhuU (∼2° along the axis perpendicular to the twofold dimer axis) (Supplementary Fig. 3a). This type of conformational change could affect the distance between coupling helices and the ATP-binding site environment in the BhuV–BhuV interface. However, the shift of the coupling helices and BhuV was also small (∼0.5 Å). We concluded that these small structural effects of BhuT-binding are consistent with the small effect of BhuT on ATPase activity.

**Figure 4:**
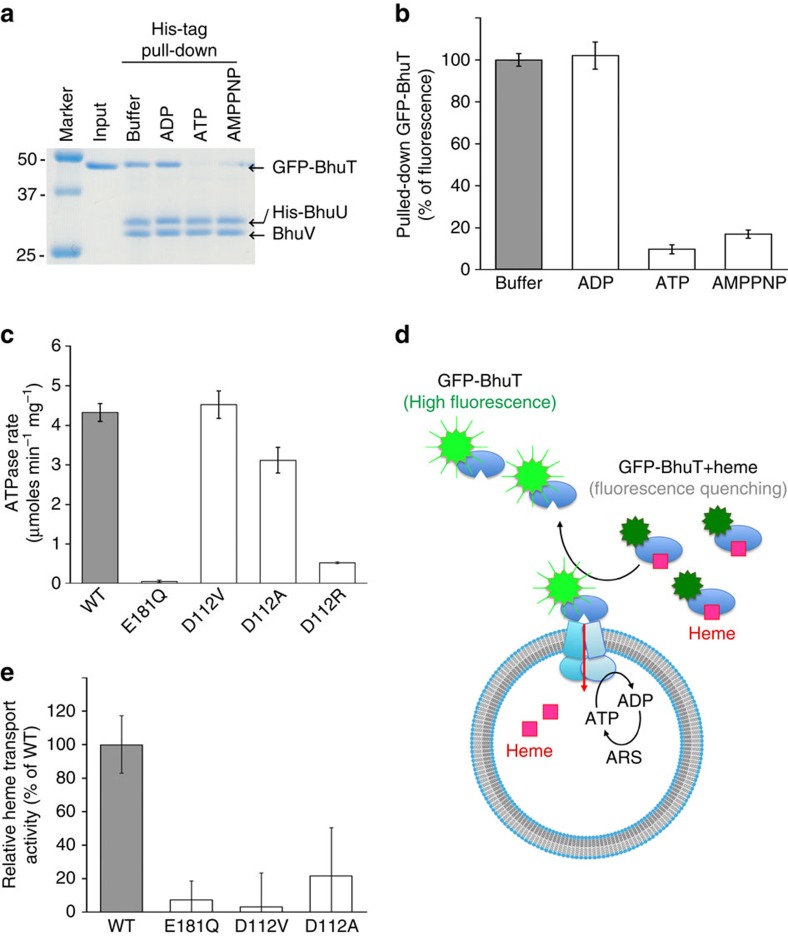
Functional analysis of the haem transporter BhuUV-T. (**a**) The BhuUV–BhuT interactions were evaluated by assessing the effect of nucleotides (ADP, ATP or ATP analogue) on the His-tag pull-down. His-tagged BhuUV was immobilized on Ni-NTA agarose resin and incubated with GFP-tagged BhuT. After washing with binding buffer (lane 3), buffer containing ADP (lane 4), ATP (lane 5) or AMPPNP (lane 6), the pulled down samples were analysed by SDS–PAGE and stained with Coomassie Brilliant Blue. A representative result is shown. (**b**) The amounts of pulled down GFP-BhuT in (**a**) are expressed as the per cent fluorescence intensity relative to the control without the nucleotide. (**c**) ATPase activity of BhuUV mutants in the detergent solution was measured to evaluate the importance of D112 in the translocating channel. E181Q is an inactive mutant in which the conserved residue in the ATPase catalytic site in BhuV is mutated. D112R was unstable during the purification owing to aggregation and showed a lower ATP hydrolysis rate. (**d**) Depiction of the transport assay protocol using BhuUV-reconstituted liposome containing an ARS. The external concentration of the haem was measured by the change of fluorescence from GFP-tagged BhuT. (**e**) Comparison of the haem transport activity. The activity is expressed in terms of the percentage of wild type (100%). Reconstitution of D112R into liposome was unsuccessful. Error bars show s.d. (*n*=3).

The effects of nucleotide (ATP or ATP analogue) binding on the BhuUV–BhuT interaction were investigated by performing pull-down experiments using His-tagged BhuUV immobilized onto Ni-NTA beads and green fluorescent protein (GFP)-tagged BhuT (Fig. 4a,b). Addition of GFP-BhuT on the immobilized BhuUV led to tight complex formation. After washing the beads with a solution containing the nucleotide ADP, ATP or non-hydrolysable ATP analogue (AMP–PNP), the sample was eluted from the beads by the buffer containing 500 mM imidazole to measure the amount of protein complex. If the ATP were the only nucleotide that could increase the dissociation of GFP-BhuT, ATP-binding would not be sufficient whereas the hydrolysis reaction would be essential for the dissociation. However, our results showed that both ATP and AMP–PNP caused the dissociation of GFP-BhuT from BhuUV. It was also confirmed that the hydrolysis product ADP does not affect the dissociation of GFP-BhuT. Therefore, we concluded that the ATP-binding to the BhuUV has a key role in reducing the affinity of BhuUV towards BhuT.

## Discussion

We prepared haem-loaded BhuT and mixed it with the bacterial ABC haem importer BhuUV to crystallize the haem importer complex BhuUV-T. However, the obtained crystals did not contain haem as judged by the colour of the crystal and the electron density. It is possible that complex formation between BhuT-haem and BhuUV during crystallization resulted in release of the haem or its translocation through the BhuU channel due to the structural flexibility of the transporter in detergent solution^6^. Following haem translocation, most of the space in the haem-binding cleft of BhuT is occupied by the H5a pair of the BhuU dimer (Fig. 3b), resulting in closure of the periplasmic gate. The structure of BhuUV-T in the haem- and nucleotide-free form was a symmetric dimer containing the inward-facing conformation of BhuU (Fig. 2).

The structure of the inward-facing symmetric dimer in the substrate- and nucleotide-free form in the reaction cycle of the haem-transporter complex allowed us to visualize the previously uncharacterized state of the type II ABC transporter. This state was predicted on the basis of crystallographic studies of the BtuCD-F (vitamin B_12_ importer) system (Supplementary Fig. 1a)^5^. According to this proposed mechanism for BtuCD, the transporter in the nucleotide-free state adopts the outward-facing conformation (state 5 in Supplementary Fig. 1a) and is transformed into another outward-facing conformation by the binding of ATP to NBD (state 1 in Supplementary Fig. 1a). Next, ATP hydrolysis and substrate translocation causes the dimeric TMD to adopt the inward-facing conformation, hitherto considered to be an energetically unstable and short-lived state. Therefore, the substrate- and nucleotide-free PBP-TMD–NBD complex in the symmetric inward-facing conformation (state 3 in Supplementary Fig. 1a) had been postulated to be a transient state from the occluded ATP-bound (state 2) to the asymmetric occluded ATP-free state (state 4 in Supplementary Fig. 1a). Our BhuUV-T structure in the inward-facing conformation in the haem- and nucleotide-free form is in the post-translocation state (state 3 in Supplementary Fig. 1b) and corresponds to the missing piece of the structures in the substrate-transport reaction cycle by type II ABC transporter. Consequently, it is now possible to discuss the haem-transfer mechanism, the conformational change of TMD and the role of ATP in the haem-transport cycle based on the present structures of haem importer BhuUV-T (Supplementary Fig. 1b).

In the first step of haem transport, the haem in BhuT would be transferred to dimeric BhuU through the periplasmic gate. Although no structure of the substrate-bound state of type II importer is available, haem-bound BhuT would likely bind to the periplasmic surface of dimeric BhuU when the periplasmic gate opens in the outward-facing conformation (state between 1 and 2 in Supplementary Fig. 1b). In this hypothetical structure, namely the haem-bound BhuT and BhuUV complex (outward-facing), the positions of the two H5a helices would be separated, based on the outward-facing conformation of HmuUV, to create an entry site for haem into the channel (Fig. 2d). The hydrophobic haem molecule would be readily transferred from the hydrophilic cavity of BhuT (Fig. 3b), through the periplasmic gate, to the hydrophobic channel of dimeric BhuU (Fig. 2a,b). Following haem transfer, the H5a helices would move into the BhuT cleft to close the periplasmic gate. Since our pull-down assay (Fig. 4a,b) showed that haem-free BhuT has high affinity for BhuUV, BhuT would continue to bind to BhuU (state 2 in Supplementary Fig. 1b) and prevent the reverse flow of haem, even after haem transfer from BhuT to BhuU.

Following this transfer, the BhuUV-T complex moves to the state after haem translocation (state 3 in Supplementary Fig. 1b), in which the periplasmic gate of BhuU is closed and its H5a interacts with the haem cavity of BhuT (Fig. 3b). On the basis of the crystal structure of BhuUV-T determined in this study, the periplasmic gate is tightly sealed by the R204-D200 salt bridges and hydrophobic interaction (L203–L203′) in H5a (Fig. 2b,c). It is suggested that H5a plays a key role as a gating helix in addition to forming the interaction site for BhuT, since mutation of the corresponding Arg residue in H5a of HmuU was reported to decrease affinity to HmuT and decrease haem-transport activity^3^.

**Figure 5:**
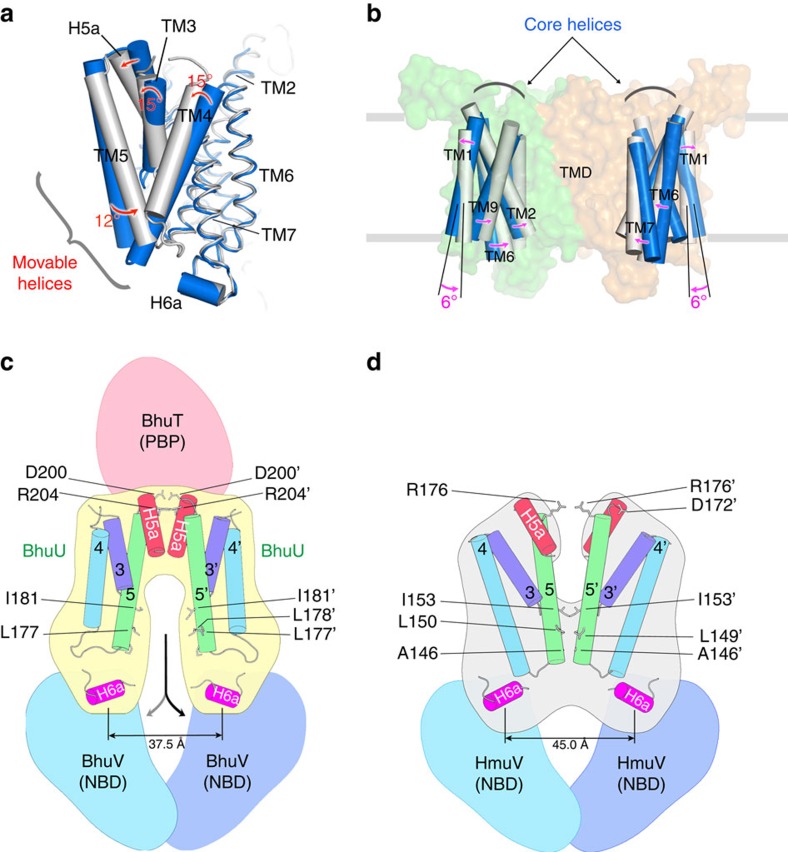
Conformational changes between the inward- and outward-facing structures of the haem transporter. (**a**) Superposition of the BhuU and HmuU monomers. The Cα atoms of the core TM helices (TM1, 2, 6–9) are used for superposition (r.m.s.d. 0.8 Å for 135 Cα atoms). TM3-5 and H5a show large deviations and are represented by cylinders for clarity. BhuU and HmuU are grey and blue, respectively. (**b**) Superposition of the BhuU dimer and HmuU dimer (r.m.s.d. 2.4 Å for 260 Cα atoms in the core TM helices). The core TM helices are shown as cylinders and TM8, TM10 and movable helices are omitted. The core TM helices can rotate as a unit by 6°. (**c**) Schematic diagram of the conformation of the movable helices (TM3-5 and H5a) in the inward-facing conformation of BhuUV-T. The distance between the H6a helices is measured from the Cα atoms of A253 in the middle of H6a. The residues involved in the periplasmic and cytoplasmic gate are represented by stick models. (**d**) Schematic diagram of the outward-facing conformation of HmuUV. The distance between the H6a helices is measured from the Cα atoms of A225 in the middle of H6a.

BhuU would be converted from the outward- to the inward-facing conformation in association with the open-to-closed change in the periplasmic gate following haem transfer into the BhuU haem-translocating channel. The structures of two haem importers, HmuUV and BhuUV, in the different conformations can now be compared. Figure 5a shows a structural alignment of the monomeric states of BhuU and HmuU. This comparison of the inward- and outward-facing conformations revealed that while the orientation of the TM1, TM2, TM6, TM7, TM8 and TM9 helices are similar (r.m.s.d. of 0.8 Å), the tilting angles of the TM3 and TM4 helices relative to the membrane plane differ by 15°, and that of TM5 also differs by 12° and has a maximum deviation of 8 Å. The former helices (TM1, TM2, TM6, TM7, TM8 and TM9) can be designated as core helices, and the latter (TM3, TM4 and TM5) as movable helices. In comparing the structures of the dimeric forms (Fig. 5b), the orientation of the core helices differs by 6° between the two conformations. These comparisons demonstrated that interconversion between the outward- and the inward-facing conformation is accompanied by rigid-body rotation of the core helices, the rotation of individual movable helices and conformational changes in the loop between these TM helices.

The different arrangement of the TM helices, and especially of TM3, TM4, TM5 and H5a, in the HmuUV and BhuUV-T complexes are schematically described in Fig. 5c,d. These structural comparisons allowed us to explore their interconversion, which should be highly related to the mechanism of bacterial haem transport. Conversion from the inward- to outward-facing conformation is expected to involve the rotation of the core helices, which alters the interaction of the BhuU–BhuU dimer interface (TM5, TM10 and H5a-H5a) and induces further conformational changes in other movable helices (TM3 and TM4) to seal the periplasmic gate and disrupt the hydrophobic interaction of TM5 at the cytoplasmic gate (Figs 2 and 3b).

When the cytoplasmic gate opening occurs, the hydrophobic gating residues (L177, L178 and I181) in TM5 of BhuU are exposed to the translocation pathway and create the narrowest point, and charged D112 is also exposed to the inward-facing translocation pathway (Fig. 2a,b,e). Interestingly, D112 is conserved in the haem importer and is the only chargeable residue in the translocating channel of BhuUV. The corresponding acidic residue D86 in the outward conformation of HmuUV is buried by transmembrane loop 7–8 between TM7 and TM8 (Fig. 2f). Exposure of D112 to the channel on opening the cytoplasmic gate should decrease the hydrophobicity of the haem translocation pathway, possibly resulting in haem secretion through the cytoplasmic gate and thus accelerating the release of the hydrophobic haem molecule from the channel. To investigate the proposed role of acidic residue D112 in the channel, we prepared the D112V, D112A and D112R mutants of BhuU and measured their ATPase hydrolysis rate and haem-transport activities using a reconstituted liposome system. As shown in Fig. 4c,e, D112V and D112A impaired transport activity, even though their level of ATPase activity was similar to that of the wild type. These results suggest that D112 is a key residue for regulating the electrostatic environment of the translocating channel and the conformational transition of TMD.

According to the mechanism proposed on the basis of the BtuCD-F studies^5^ (Supplementary Fig. 1a), three events are expected following substrate transport: the conformational change from the inward- to the outward-facing state; the dissociation of substrate-free PBP from the transporter complex in the post-translational state; and the binding of ATP to NBD. These processes are necessary steps for the transporter to turn over the transport cycle, but the order of each step has been unclear. Our results show that the inward-facing conformation of BhuUV-T is a stable conformation of the nucleotide-free state. There are examples of spectroscopic characterization of channels and transporters with conformational populations of the TM helices that are different in lipid bilayers from those in detergent solution^21^,^22^,^23^,^24^,^25^. The different conformations observed in crystal structures of HmuUV and BtuCD also indicate that, even in the same subfamily of ABC importers, the stability of a state depends on the type of transport substrate and organism species. Although we should interpret the structures in detergent micelles carefully, our structural analysis showed that, in the absence of ATP, BhuUV without BhuT is still in the inward-facing conformation, indicating that BhuT dissociation is not a critical factor that imposes the conformational state from the inward- to the outward-facing conformation and initializes the haem-transport reaction.

Moreover, as shown in Fig. 4, our pull-down assay results demonstrated that BhuT was very tightly bound to BhuUV in the absence of nucleotide, but the affinity of BhuT was decreased by the addition of ATP or ATP analogue. Therefore, in our proposed haem transporter mechanism, ATP-binding facilitates the dissociation of haem-free BhuT from BhuUV after transport of the haem (route a in Supplementary Fig. 1b), and eventually converts the BhuU conformation from the inward- to the outward-facing state. In the case of BtuCD-F, two routes (a and b in Supplementary Fig. 1a) through an asymmetric occluded state are proposed^5^. In this mechanism, BtuF dissociates from the asymmetric occluded state before or after ATP-binding to BtuCD. The asymmetric conformation of the TMD dimer found in the crystal structure of BhuCD-F (PDB code 2QI9)^7^ is proposed as an intermediate from the inward- to outward-facing state. It is unclear whether BhuUV-T could also adopt such an asymmetric conformation in the intermediate state, because BhuUV structures in the present study have symmetric TMD dimers. Since BhuUV-T is a very tight complex and the cytoplasmic concentration of ATP is in the millimolar range, formation of the ATP-bound intermediate (state 4′ of route a in Supplementary Fig. 1b) should be a more plausible event after the post-translocation state (state 3) in living cells. Therefore the nucleotide-free BhuUV in route b is unlikely under physiological conditions.

Turning next to the TMD–NBD complex of type II ABC transporter in the nucleotide-free form, both BhuUV and MolAB^4^ (33% amino-acid identity in TMD) adopt similar inward-facing conformations (rmsd of 1.1 Å for 205 Cα atoms in TM helices; Supplementary Fig. 5), whereas another haem importer, HmuUV (45% amino-acid identity in TMD; r.m.s.d. of 1.7 Å for 185 Cα atoms) and BtuCD (37% amino-acid identity in TMD; rmsd of 1.9 Å for 191 Cα atoms) are in the outward-facing conformation. The stability of each conformation of type II importer likely differs by bacterial source or substrate type in the nucleotide-free form.

We successfully characterized the structures of the ABC haem importer, the Bhu system from *Burkholderia cenocepacia*, and showed that both BhuUV-T and BhuUV adopt an inward-facing conformation in which the periplasmic gate of the haem-translocating channel is closed and the cytoplasmic gate is open. Structural comparison of the Bhu system with another haem importer, the Hmu system, showed that interconversion of the haem-translocating channel from the outward- to the inward-facing conformation is accompanied by a change in the polarity of the channel, promoting secretion of the hydrophobic haem molecule into the cytoplasm. In addition, ATP-binding to BhuV is responsible for the dissociation of tightly bound BhuT from BhuU. Conversion to the outward-facing conformation likely restores the transporter to the initial state of the haem-import cycle. This proposed mechanism appears to be consistent with a coupling mechanism proposed for type II ABC importer BtuCD-F (vitamin B_12_ transporter)^5^. Although the details regarding substrate transport vary among the ABC transporters, the role of ATP-binding in the conformational change of NBD and TMD (inward- to outward-facing) is likely to be common among the ABC-exporters and type I (for example, MalFGK_2_ (ref. 26)) and type II importers. The effect of ATP-binding on PBP affinity also appears to be common in the type I importer families.

## Methods

### Protein expression

BhuU and BhuV were expressed from a single plasmid. The genes encoding full-length BhuU (with an N-terminal 8 His-tag and enterokinase cleavage site) and BhuV were cloned into the pET-19b vector (Merck Millipore). *E. coli* c41 (DE3) cells were transformed with the plasmid and grown in LB medium containing 50 μg ml^−1^ ampicillin at 37 °C. Overexpression of His-BhuUV was induced with 0.3 mM IPTG at an OD_600_ of 0.6–0.8. The cells were grown for a further 20 h at 16 °C. BhuT was expressed in the cytoplasm of *E. coli* Rosetta2 (DE3) cells (Merck Millipore). Since N-terminal 33 residues of BhuT were predicted as a signal sequence by SignalP^27^, cDNA-encoding residues 40–311 of BhuT were cloned into the pGEX-6P-1 vector (GE Healthcare) using the *Bam*HI and *Eco*RI sites. The N-terminal of the BhuT protein was fused to a glutathione *S*-transferase (GST) tag with PreScission protease cleavage site (GE Healthcare). The transformed cells were cultured in LB medium (1 L) containing 50 μg ml^−1^ ampicillin and 34 μg ml^−1^ chloramphenicol at 37 °C, and overexpression of GST-BhuT was induced with 0.3 mM IPTG at an OD_600_ of 0.6–0.8. The cells were grown for a further 20 h at 16 °C. The cDNA of BhuT fused to the GFP-tag at the N-terminal was cloned into the pET19b vector. The GFP-tagged BhuT was expressed in Rosetta2 (DE3) cells using the same procedure as for GST-BhuT. The sequences of the primers used in the work are listed in Supplementary Table 1.

### Purification of BhuUV

Cells (typically 18 g from a 4 l cell culture) were resuspended in lysis buffer (50 mM Tris-HCl pH 7.5, 150 mM NaCl) with 2 mM MgCl_2_, 10 μg ml^−1^ DNase, 0.2 mg ml^−1^ lysozyme and one tablet of cOmplete EDTA-free (Roche) and disrupted in a French-press (Ohtake, Tokyo). Cell debris was removed by centrifugation using an R13A angle rotor and CR22N centrifuge (Hitachi) at 7,400*g* for 30 min. The supernatant was collected and ultracentrifuged using a P45AT angle rotor and CP80WX ultracentrifuge (Hitachi) at 66,000*g* for 1 h to obtain the membrane fraction. Collected membranes were stored at −80 °C. For purification, membranes were solubilized in lysis buffer with the addition of 2% (w/v) *n*-decyl-β-D-maltopyranoside (DM; Dojindo) for 1 h at 4 °C. The insoluble fraction was separated by centrifugation using the P45AT angle rotor at 66,000*g* for 30 min. Solubilized membranes were loaded onto Ni-NTA agarose resin (Qiagen) in a chromatography column (Bio-Rad) and washed extensively with buffer A (lysis buffer containing 0.15% DM and 50 mM imidazole). BhuUV protein was eluted with buffer A containing 300 mM imidazole. The eluted protein was desalted on a HiPrep desalting column 26/10 (GE Healthcare) in buffer containing 50 mM Tris-HCl pH 8.5, 20 mM NaCl and 2 mM MgCl_2_, then further purified by anion-exchange chromatography (HiTrap Q HP, GE Healthcare). The eluted fraction was collected and concentrated using an Amicon Ultra filter (Merck Millipore; 50 kDa cutoff).

### Purification of BhuT

Collected cells expressing GST-tagged BhuT were suspended in lysis buffer (50 mM Tris-HCl pH 7.6, 150 mM NaCl) and disrupted in a French press. The lysate was centrifuged at 66,000*g* for 1 h using a P45AT rotor to remove cell debris. The supernatant was loaded onto a glutathione Sepharose 4B resin (30 ml; GE Healthcare) column. The resin was washed with lysis buffer and eluted with 10 mM reduced glutathione in 50 mM Tris-HCl pH 7.6 and 150 mM NaCl. The eluted fraction was mixed with PreScission protease (GE Healthcare) and dialysed against 50 mM Tris-HCl pH 7.6, 150 mM NaCl and 1 mM DTT. After cleavage of the GST-tag, the BhuT sample was dialysed against 50 mM Tris-HCl pH 7.6 and 1 mM DTT, then applied to an anion-exchange chromatography column (Source 15Q; GE Healthcare), and the flow-through fraction was collected. The sample was concentrated using an Amicon Ultra filter (10 kDa cutoff, Merck Millipore) to 10 mg ml^−1^ for assays and crystallization. The protein concentration was determined using a bicinchoninic acid assay kit (Pierce).

GFP-tagged BhuT was purified by applying the cell lysate supernatant after ultracentrifugation onto a DEAE Sepharose Fast Flow (60 ml; GE Healthcare) column and eluted with a linear gradient of NaCl (0-1.0 M) in 50 mM Tris-HCl pH 8.0 and 1 mM EDTA. The fractions containing GFP-BhuT were dialysed against 40 mM HEPES pH 7.0 and 1 mM EDTA and applied to a Source 15 S column (GE Healthcare). The bound protein was eluted with a linear gradient of NaCl (0–0.5 M). The peak fraction was collected and concentrated using an Amicon Ultra filter (50 kDa cutoff).

### Preparation of the BhuUV-T complex

Membranes containing BhuUV were resuspended in lysis buffer (50 mM Tris-HCl pH 7.5, 150 mM NaCl) plus 2 mM MgCl_2_ and solubilized by the addition of 1.5% (w/v) *n*-nonyl-β-D-glucopyranoside (NG; Anatrace) for 1 h at 4 °C. The insoluble fraction was removed by centrifugation at 66,000*g* using a P45AT angle rotor for 30 min. Solubilized membranes were loaded onto Ni-NTA agarose resin (10 ml; Qiagen) packed in a chromatography column (Bio-Rad) and washed extensively with buffer B (lysis buffer with 2 mM MgCl_2_ and 0.34% NG) containing 50 mM imidazole. Purified BhuT (5 ml, 2 mg ml^−1^) supplemented with 0.1 mM hemin was applied to a BhuUV-bound Ni-NTA column. The resin was washed with buffer B containing 50 mM imidazole. BhuUV-T complex was eluted from the resin using buffer B containing 300 mM imidazole. The eluted protein was concentrated and applied to a size-exclusion chromatography column (Superdex 200 GL 10/30, GE Healthcare) equilibrated with buffer B. The peak fraction of BhuUV-T was collected and concentrated to 26 mg ml^−1^ for crystallization.

### Crystallization and X-ray data collection

BhuUV-T was crystallized at 20 °C using the sitting-drop vapour diffusion method. The drop was formed by mixing the protein with reservoir solution containing 15% PEG 2000 and 0.1 M HEPES pH 7.6 in a 1:1 ratio. Colourless crystals appeared after 3 days. For X-ray data collection at 100 K, crystals were cryoprotected by soaking in increasing concentrations of PEG 2000 from 16 to 28% in 2% steps and then flash-frozen in liquid nitrogen. BhuUV was crystallized using a protein concentration of 15 mg ml^−1^ and a reservoir solution containing 30% PEG 400, 0.1 M NaCl, 0.1 M Li_2_SO_4_ and 0.1 M Tris-HCl pH 8.5 (4 °C). The crystal was flash-frozen in liquid nitrogen. X-ray diffraction data were collected using a wavelength of 1.0 Å at BL26B2 and BL41XU in SPring-8, Japan, and processed using HKL2000 (ref. 28) and the CCP4 program suite^29^. Data collection and refinement statistics are shown in Table 1.

### Structure determination and refinement

The initial phase of the BhuUV-T crystal was obtained using the molecular replacement (MR) method. The structure of BtuCD-F (PDB code 2QI9) was modified using the program Chainsaw^30^ by pruning the non-conserved residues to prepare the search model for MR. The MR phase obtained using the program Phenix^31^ was improved using the program DM^32^, and the protein model was manually rebuilt using the Coot program^33^. The electron density of the BhuT region was very poor and model building was based on the BhuT structure determined separately. The atomic coordinates of BhuUV-T were further refined with multiple rounds of manual rebuilding followed by restrained refinement using Phenix^31^.

Non-uniform binding of BhuT was suspected based on the poor electron density and the example of the crystal structure of BtuCD-F. The fact that the BhuT molecule in the complex is free from crystal packing contact also increased the possibility of a mixture of two orientations in the crystal. We placed the second orientation of BhuT by rotating 180° along the dimeric axis of BhuUV and refined with an occupancy ratio of 0.6:0.4. The free *R* after the refinement of this model was slightly lower (31.4%) than that of original model with one orientation of occupancy (31.8%). However, the electron density for the second molecule was still not clearly identified. Therefore, we refined the final model with only major orientation of BhuT with a fixed occupancy of 0.6, because it yielded the lowest free *R* (31.0%). In the final model, 27% of BhuT residues, mostly in the loop region, showed real space correlation coefficients^31^ of <0.6, suggesting that the fit of model to density was poor for these residues. However, the geometries of the model evaluated by MolProbity^34^ were excellent: Ramachandran plot analysis of the main chain of BhuT region showed that 98% were in favoured regions. Rotamer analysis of the side-chain (chi1–chi2 plot) showed that 98% of residues lie in the favoured regions.

In the structure analysis of the BhuUV, the initial phase of the X-ray data was obtained by MR using the model from BhuUV-T. The coordinates of BhuUV were refined by multiple rounds of manual rebuilding using Coot^33^ followed by restrained refinement using Phenix^31^. The statistics for the structure refinement are summarized in Table 1. The SSM program^35^ was used for the structural superposition calculations.

### Pull-down assay

All experiments were performed on ice and the solutions were prechilled. Ni-NTA agarose resin (50 μl) in a Micro Bio-Spin Column (Bio-Rad) was equilibrated with binding buffer (50 mM Tris pH 7.5, 150 mM NaCl, 1 mM MgCl_2_ and 0.15% DM). Purified BhuUV (0.3 ml, 0.12 mg ml^−1^) in binding buffer was applied to the column. The resin was washed four times with washing buffer (0.3 ml, binding buffer with 50 mM imidazole) and binding buffer (0.3 ml). Purified GFP-labelled BhuT (0.3 ml, 10 μg) was applied to BhuUV-bound Ni-NTA resin. The resin was washed four times with washing buffer (0.3 ml). The resin was incubated for 5 min in solution (0.3 ml) containing 1 mM ADP, ATP or AMP–PNP, then the unbound GFP-BhuT was removed by washing the resin with the same solution (0.3 ml). The resin was washed twice with washing buffer (0.3 ml). The protein was eluted from the resin using the binding buffer (0.3 ml) containing 500 mM imidazole. The amount of GFP-BhuT complexed with BhuUV in the eluted fractions was visualized by SDS–PAGE and measured by the GFP fluorescence with a fluorescence spectrophotometer (F-7000, Hitachi). The uncropped SDS–PAGE gel is shown in Supplementary Fig. 6.

### ATPase activity assay

ATPase activity was measured by determining the concentrations of inorganic phosphate. The reaction solution contained buffer C (50 mM Tris-HCl pH 7.5, 150 mM NaCl, 10 mM MgCl_2_ and 0.15% DM) with 14 nM BhuUV. After incubation for 10 min at 37 °C, various concentrations of ATP were added to the sample to initiate the reaction (total volume of 200 μl). Aliquots of reaction solution (40 μl) were removed at various times and mixed with 12% SDS (40 μl) to stop the reaction. ATPase activity in the presence of BhuT was measured as above except that 0–5 μM BhuT was present. The concentration of inorganic phosphate was determined by a molybdenum-based standard protocol^36^. Briefly, colour was developed for 3–10 min following the addition of buffer D (50 μl) consisting of 6% ascorbic acid (25 μl) in 1 N HCl and 1% ammonium molybdate (25 μl). The reactions were stopped by the addition of solution E (75 μl; 2% sodium citrate, 2% sodium meta-arsenite and 2% acetic acid) and read at 850 nm using a microplate reader (SpectraMax 190, Molecular Devices). All experiments were run at least three times. All data analyses were performed using IGOR Pro 6 (WaveMetrics).

### Reconstitution of BhuUV into liposomes

Liposomes were prepared using a protocol as described previously^3^,^18^, with minor modifications. *E. coli* polar lipid (powder, Avanti Polar Lipids) and egg yolk L-α-phosphatidylcholine (20 mg ml^−1^ in chloroform, Avanti Polar Lipids) were mixed at a ratio of 3:1 (w/w) and the chloroform was removed by rotary evaporator. Dried lipids were resuspended in 50 mM Tris-HCl 7.5, 150 mM NaCl and 2 mM MgCl_2_ at a concentration of 20 mg ml^−1^ and sonicated using a micro tip (Sonifier 450, Branson). The lipid suspension was stored at −80 °C until use.

For the reconstitution of the transporter, purified wild type or mutant BhuUV (final 0.1 mg ml^−1^) in 50 mM TrisHCl pH 7.5, 150 mM NaCl, 2 mM MgCl_2_ and 0.15% DM was added to the liposomes (final 10 mg ml^−1^) destabilized by 0.15% DM. After the incubation at 4 °C for 1 h with gentle rotation, the detergent was removed by addition of 40 mg ml^−1^ Bio-Beads SM-2 (Bio-Rad) resin at 0, 15, 40 and 90 min, and then further incubated at 4 °C overnight. The resin was removed by centrifugation. The reconstituted proteoliposomes were extruded through a 400 nm polycarbonate membrane filter (Mini-Extruder, Avanti Polar Lipids) and collected by ultracentrifugation (CP-80MX with a P50A3 rotor, Hitachi) at 117,000*g* for 15 min. The ratio of transporter facing right-side-in and inside-out was estimated to be 50:50 under these conditions by SDS–PAGE analysis. To measure the effect of BhuT on the ATPase rate of the transporter orientated in the inside-out direction, BhuT was incorporated into the proteoliposomes by three freeze–thaw cycles in the buffer containing BhuT. The proteoliposomes were rinsed three times by ultracentrifugation.

For the transport assay, the proteoliposome pellet was resuspended in the buffer containing 50 mM TrisHCl pH 7.5, 150 mM NaCl, 2 mM MgCl_2_ and the ATP-regeneration system (ARS: 10 mM ATP, 100 mM phosphoenolpyruvate, 0.1 U ml^−1^ pyruvate kinase). ARS was incorporated into the proteoliposomes by three freeze–thaw cycles. The proteoliposomes were harvested by ultracentrifugation for 15 min at 117,000*g* and rinsed three times with resuspension buffer (50 mM TrisHCl pH 7.5 and 150 mM NaCl). The suspension was kept on ice to limit the depletion of ATP by the basal ATPase activity of BhuUV before the transport assay.

### *In vitro* transport assay

Aliquots (50 μl) of ARS-incorporated proteoliposomes were incubated at 37 °C for 5 min and hemin-bound GFP-BhuT was added to initiate the transport reaction. The final concentrations of the assay mixture were 17.5 mg ml^−1^ lipid, 2.6 μM BhuUV, 15 μM BhuT and 15 μM hemin. A microtube containing the assay mixture was placed on ice at various time points (0–10 min) to stop the reaction. The proteoliposomes in the aliquots were rapidly pelleted by ultracentrifugation at 4 °C, 117,000*g* for 15 min and the supernatant was collected to measure the haem concentration.

The binding of the haem to GFP-BhuT quenches the intensity of the fluorescence. Therefore, as illustrated in Fig. 4d, the decrease of the haem in the external solution was detected by measuring the increase in fluorescence from the GFP-BhuT. The supernatant (40 μl) of the transport assay mixture was diluted with 50 mM Tris HCl pH 7.5 and 150 mM NaCl (80 μl) to measure the fluorescence (F-7000, Hitachi; excitation at 488 nm; emission at 508 nm) in microcuvettes.

### Amino-acid sequences

GenBank accession codes for the proteins used for the sequence comparison of the haem transporters in Fig. 3 are: Bc_BhuU, WP_012493591; Yp_HmuU, WP_002209059; Sd_ShuU, YP_405014; Bp_BhuU, NP_879217; Pa_PhuU, WP_003121063; Sm_HmuU, NP_386536; Vc_HutC, NP_233299; Pd_HutC, ZP_06155495. The ConSurf server was used for the sequence comparison^37^. The other type II ABC importers for the alignment in Fig. 3 are: Ec_BtuC, NP_416226; Ec_FecC, NP_418709; Hi_MolB, NP_439622.

### Data availability

Coordinates and structure factors have been deposited in the Protein Data Bank under accession codes 5B57 (BhuUV) and 5B58 (BhuUV-T). GenBank accession codes used for sequence comparison are presented in the Methods section “Amino-acids sequences”. All additional experimental data are available from the corresponding author on reasonable request.

## Additional information

**How to cite this article**: Naoe, Y. *et al*. Crystal structure of bacterial haem importer complex in the inward-facing conformation. *Nat. Commun.* **7**, 13411 doi: 10.1038/ncomms13411 (2016).

**Publisher's note:** Springer Nature remains neutral with regard to jurisdictional claims in published maps and institutional affiliations.

## Supplementary Material

Supplementary Information Supplementary Figure 1-6 and Supplementary Table 1.

## Figures and Tables

**Table 1:** Data collection and refinement statistics.

	**BhuUV-T**	**BhuUV**
*Data collection*		
Space group	*P*2_1_2_1_2_1_	*P*2_1_2_1_2_1_
Cell dimensions		
*a*, *b*, *c* (Å)	69.02, 95.75, 253.50	110.82, 118.28, 142.75
Resolution (Å)	50–3.2 (3.26–3.20)*	50–2.8 (2.85–2.80)*
*R*_sym_ (%)	9.2 (43.8)	10.9 (87.8)
*I*/σ (*I)*	10.0 (1.8)	16.5 (1.4)
CC^1/2^	(0.539)	(0.732)
Completeness (%)	96.9 (92.6)	99.1 (92.5)
Redundancy	3.9 (2.5)	7.6 (5.9)
Wilson *B* (Å^2^)	61.7	40.5
		
*Refinement*		
Resolution (Å)	50–3.2	50–2.8
No. reflections	22421	38130
*R*_work_/*R*_free_	0.272/0.310	0.212/0.268
No. of atoms		
Protein	10446	8633
Ligand/ion	0	76
Water	0	22
*B*-factors (Å^2^)		
Protein	75.4	43.4
Ligand/ion	—	66.8
Water	—	29.3
Coordinate error (Å)†	0.46	0.38
r.m.s. deviations		
Bond lengths (Å)	0.009	0.007
Bond angles (°)	1.1	1.2

r.m.s., root mean square.

^*^Values in parentheses are for the highest-resolution shell. One crystal was used for each data set.

^†^Maximum likelihood-based coordinate error estimate from phenix.refine^31^.
