# Supplementary material for: Crystal structure of bacterial haem importer complex in the inward-facing conformation
Source: Nat Commun. 2016 Nov 10;7:13411. doi: 10.1038/ncomms13411 (PMC5136619; doi:10.1038/ncomms13411)
Supplement: Supplementary Information — Supplementary Figure 1-6 and Supplementary Table 1. [file ncomms13411-s1.pdf]

Supplementary Information

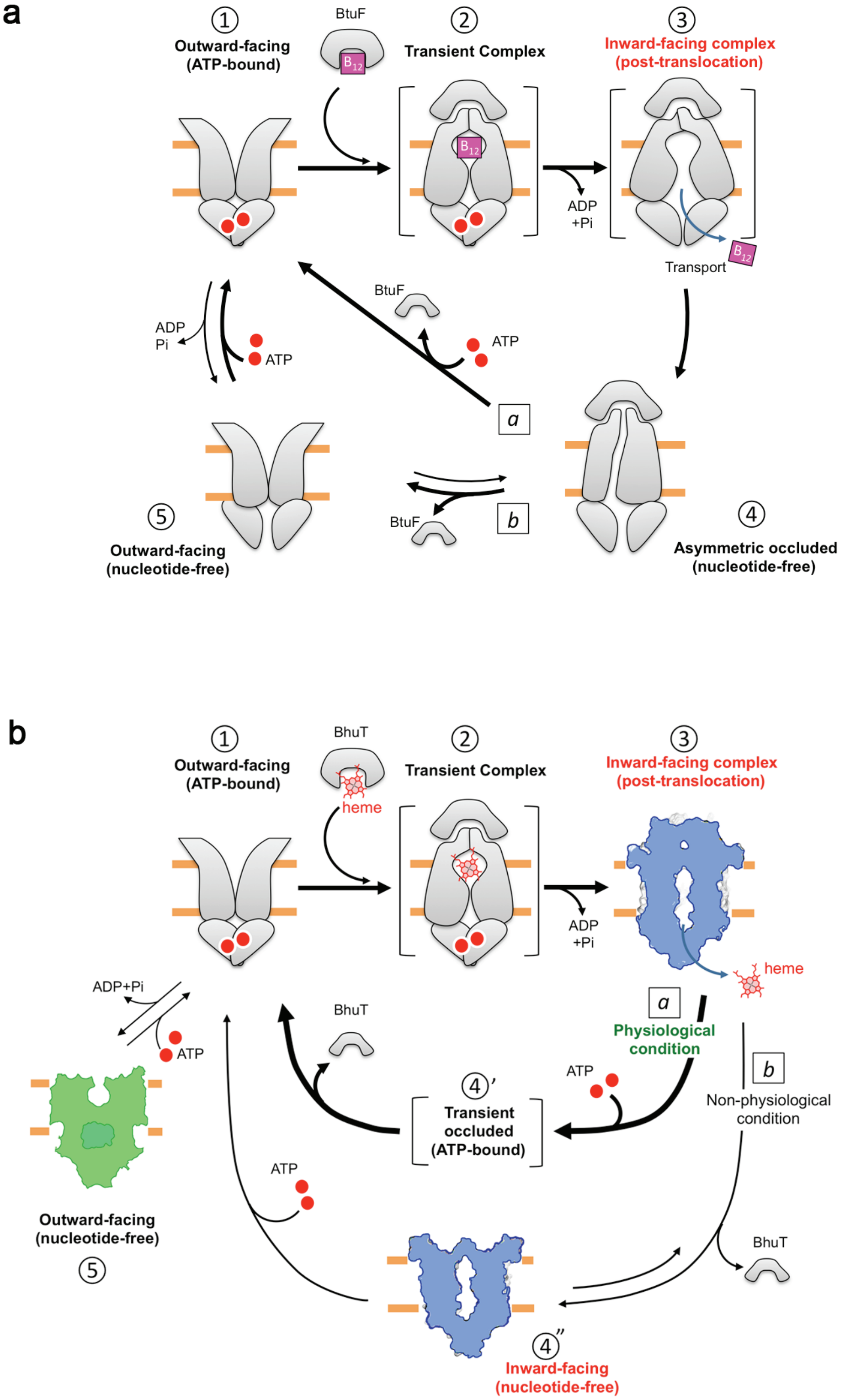

Supplementary Figure 1 | Proposed mechanism of the type II ABC transporters.

(**a**) The mechanism proposed for vitamin B_{12} transporter BtuCD-F starts with the outward-facing conformation, with ATP bound to NBD (state 1), and is based on the BtuCD structure (PDB 4R9U). Upon binding of a substrate-bound PBP to the TMD dimer, substrate (vitamin B_{12}) is released into the pocket of

the TMD dimer (state 2). The ATP molecules in the catalytic site of NBD are hydrolyzed, resulting in the helices rearranging to adopt the inward-facing conformation (state-3, post-translocation state). No structure is available for the intermediate complex (state 2) and state 3 for BtuCD-F. The asymmetric occluded state (state-4) is observed in BtuCD-F (PDB 2QI9), and routes a and b are proposed for BtuCD-F. State 5 is based on the nucleotide-free outward-facing conformation of BtuCD (1L7V). (**b**) The translocation cycle of the heme importers based on the available crystal structures. States 1 and 2 are based on the proposed mechanism for BtuCD-F. The post-translocation state (state 3) is observed for the first time in the structure of BhuUV-T solved in the present study. Since BhuUV-T is a very tight complex and the cytoplasmic concentration of ATP is in the millimolar range, route a, in which ATP-binding facilitates the dissociation of heme-free BhuT, should be preferred in living cells. The inward-facing nucleotide-free form (state-4") in route b is based on the structures of BhuUV (solved in this study) and MolAB (PDB 2NQ2), whereas this route might not be physiologically relevant. The outward-facing conformation in the nucleotide-free form (state 5) is based on HmuUV (PDB 4G1U).

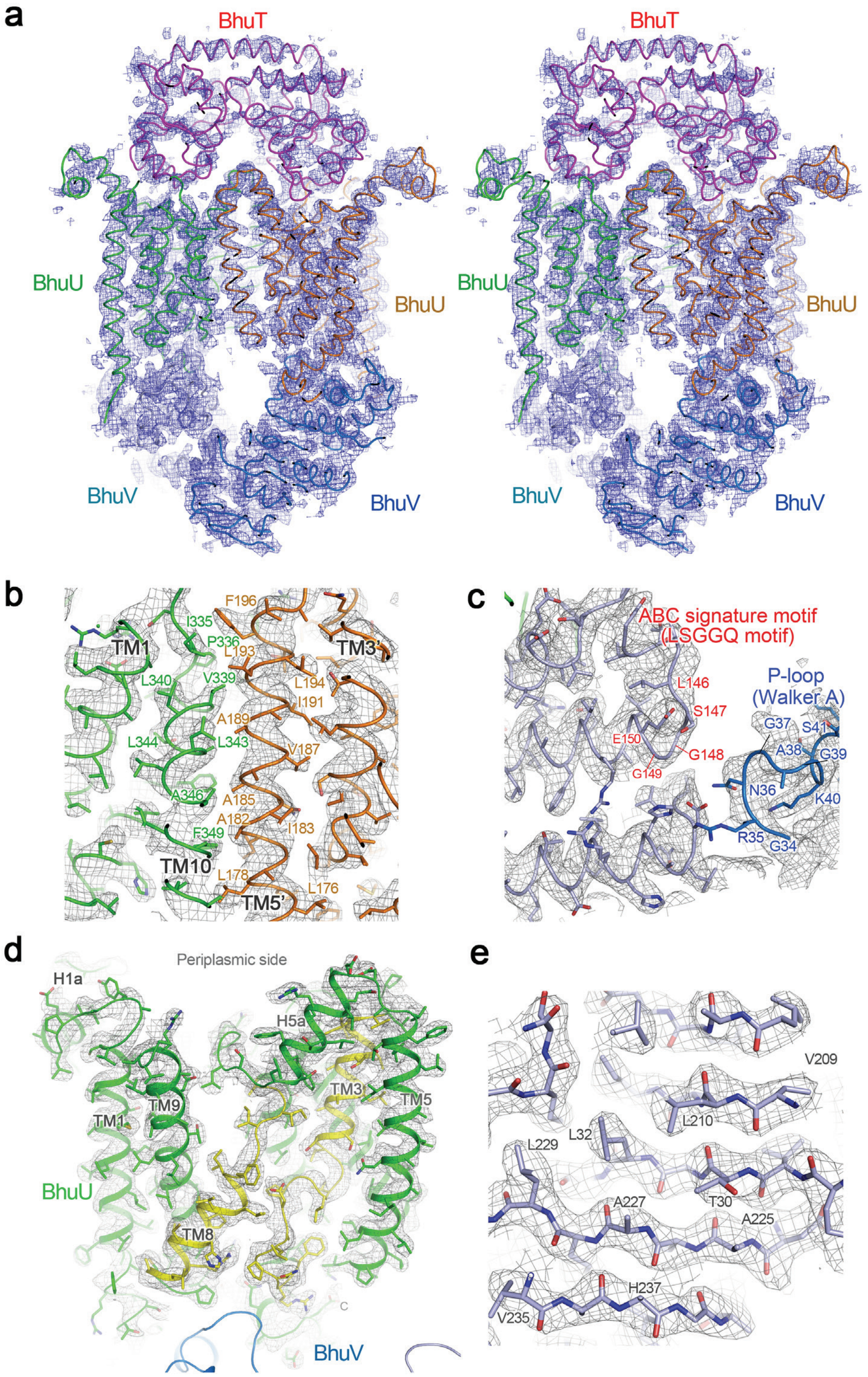

Supplementary Figure 2 | Quality of the electron density map of BhuUV-T and BhuUV.

(**a**) Stereo view of composite omit map of the BhuUV-T complex is contoured at 1.0 σ using 3.2 Å resolution data. (**b**) $2F_o$-F_c electron density map of the dimer interface of TMD of BhuU in the BhuUV-T complex. (**c**) $2F_o$-F_c map around the ABC signature sequence (LSGGQ/E) and P-loop (or Walker A) motif (GXXGXGKS/T) in the BhuUV-T complex. (**d**) $2F_o$-F_c map of TMD in BhuUV using 2.8 Å resolution data. (**e**) $2F_o$-F_c map of the β-sheet in the NBD of BhuUV.

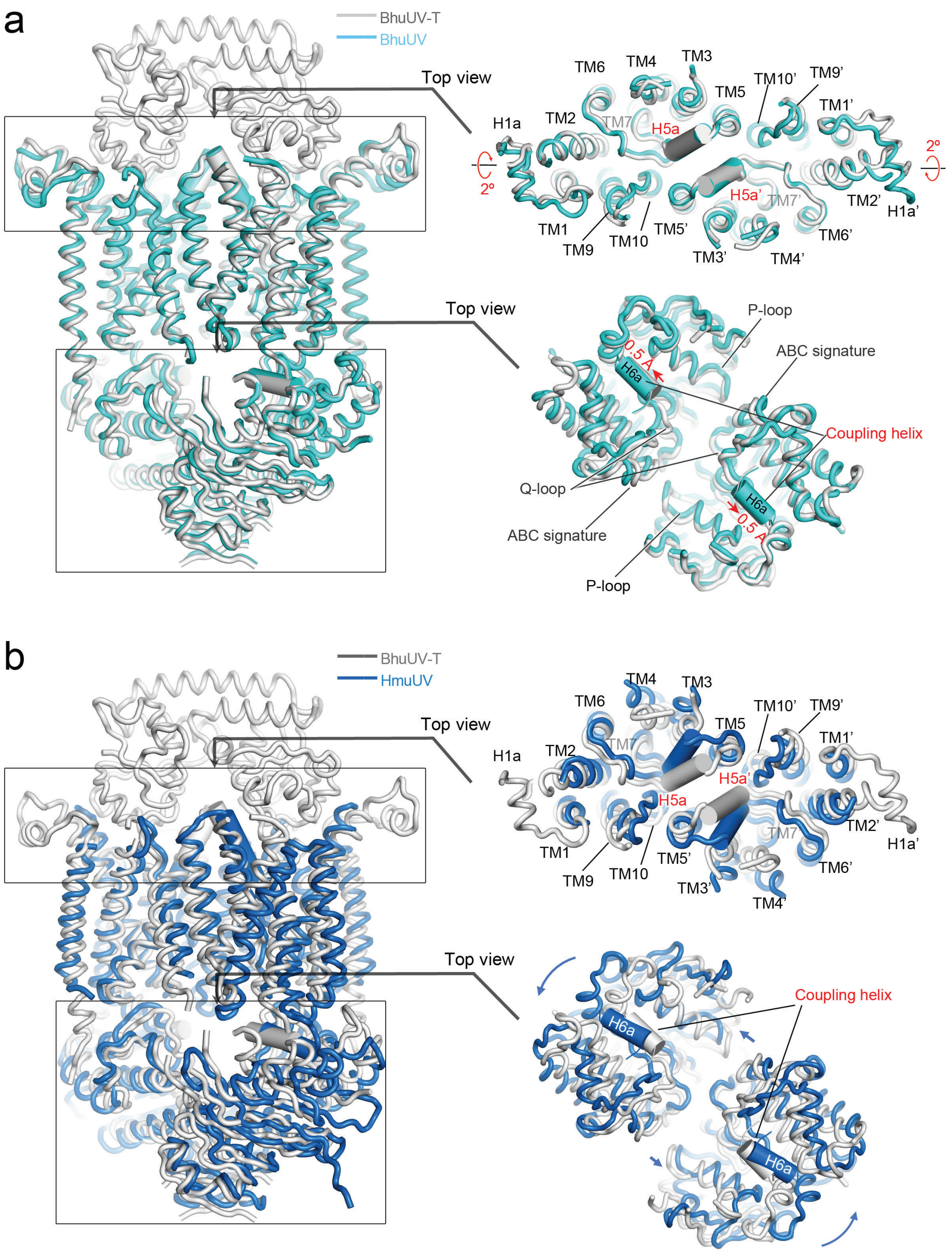

Supplementary Figure 3 | Structural superposition of heme transporters. (**a**) Superposition of BhuUV-T (gray) and BhuUV (cyan). The periplasmic gating helices H5a and coupling helices H6a of dimeric TMD are represented by cylinders for clarity. In the right upper panel, the arrangement of TM helices around the periplasmic gate is viewed from the periplasmic side and BhuT is omitted. In the right bottom panel, the dimeric NBD and the coupling helices of TMD are shown viewed from the periplasmic side. BhuUV shows a slightly longer distance between two NBDs. (**b**) Superposition of BhuUV-T (gray) and HmuUV (blue).

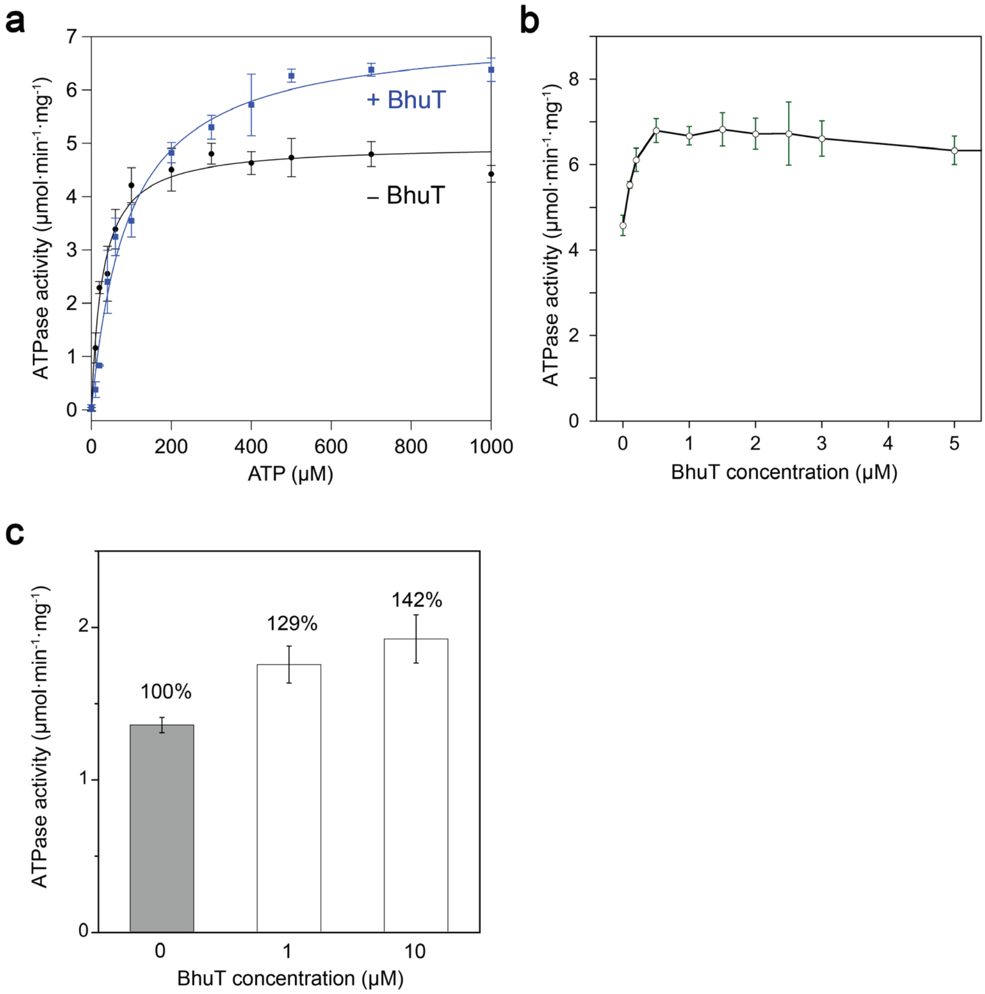

Supplementary Figure 4 | ATPase activity of the purified proteins.

(**a**) ATPase rates of BhuUV (black) and the effect of 1 μM BhuT (blue) in detergent solution (0.15% n-decyl-β-D-maltopyranoside). (**b**) ATPase rates of BhuUV as a function of BhuT concentration measured in detergent solution at 1 mM ATP. The concentration of BhuUV was 5 μg ml^{-1} (74 nM) in the reaction solution. (**c**) Effect of BhuT on ATPase rate of BhuUV reconstituted in the liposome. The activity were measured for inside-out orientation of transporter (50% of total transporter at 5 μg ml^{-1}). The concentration of BhuT during the encapsulation in proteoliposomes by freeze-thaw process are indicated. In all panels, data is the average of four replicates. The error bars represent the standard deviation.

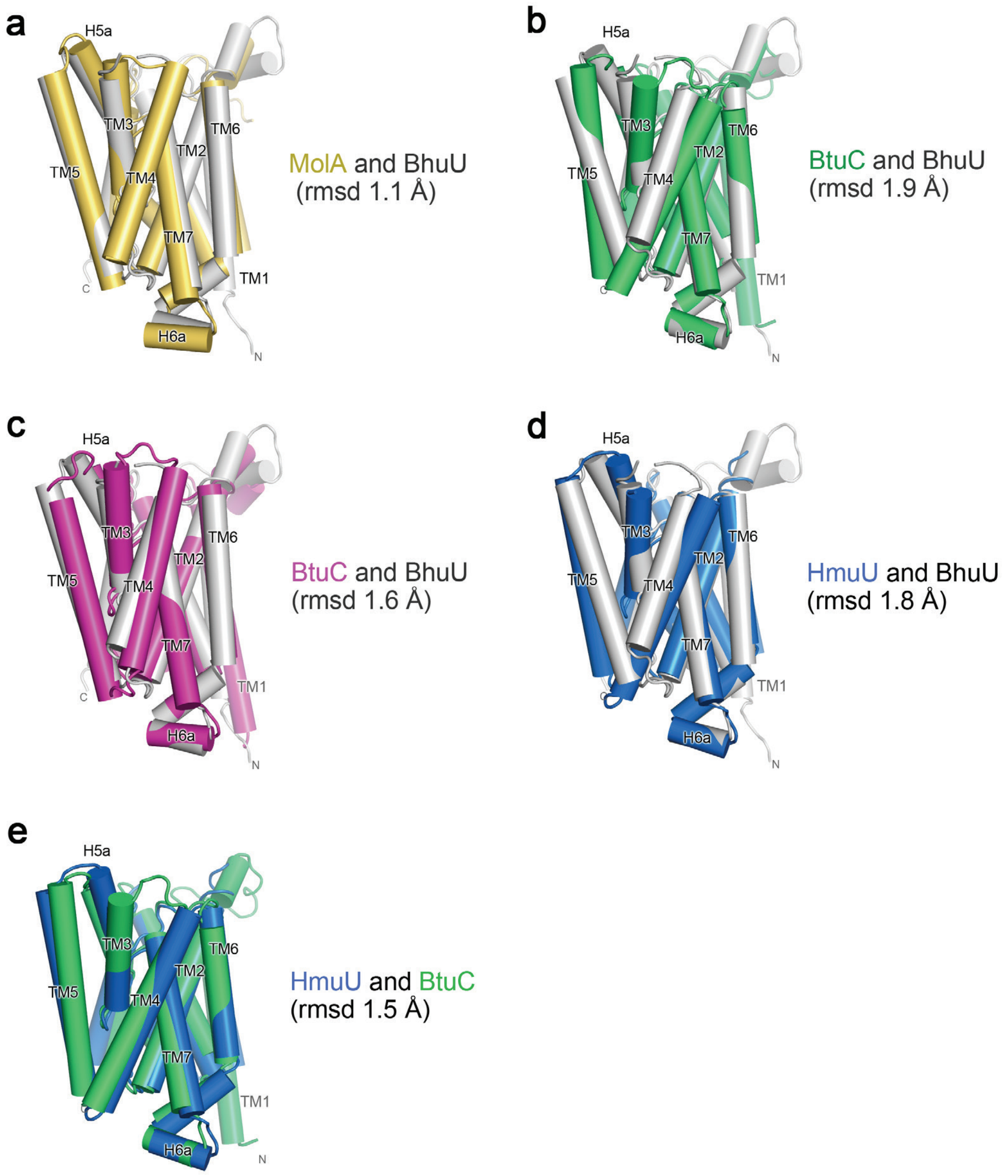

Supplementary Figure 5 | Comparisons of the conformations of the TMD subunit of type II subfamily of ABC importers. (**a**) Superposition of the monomers of the transmembrane (TM) subunit of molybdate transporter in inward-facing conformation (MolAB, PDB code 2NQ2) and heme transporter BhuUV (this study). Rmsd is 1.1 Å for 205 Cα atom pairs of the TM helices. (**b**) Superposition of the monomers of the TM subunit of vitamin B_{12} transporter (nucleotide-free BtuCD, PDB code 1L7V) and heme transporter BhuUV. Rmsd is 1.9 Å for 191 Cα atom pairs of the TM helices. (**c**) Superposition of the monomers of the TM subunit of vitamin B_{12} transporter (AMP-PNP-bound BtuCD; PDB code 4R9U) and heme transporter BhuUV. Rmsd is 1.6 Å for 208 Cα atom pairs of the TM helices. (**d**) Superposition of the monomers of the transmembrane (TM) subunit of heme transporter HmuUV (PDB code 4G1U) and heme transporter BhuUV. Rmsd is 1.8 Å for 185 Cα atom pairs of the TM helices. (**e**) Superposition of the monomers of the TM subunit of vitamin B_{12} transporter BtuCD-F (PDB code 1L7V) and TM subunit of heme transporter HmuUV. Rmsd is 1.5 Å for 214 Cα atom pairs of the TM helices.

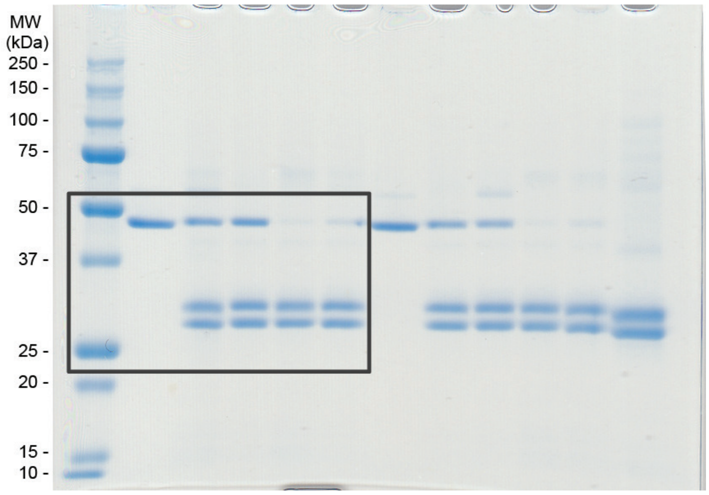

Supplementary Figure 6 | Uncroppped image of SDS-PAGE for pull-down assay of BhuUV and GFP-BhuT in Figure 4a. The first left lane is the molecular marker.

Supplementary Table 1 | The primers used in this work

Primer	Sequence
GST-BhuT Forward 1	GATCCAAGCGCGTGATTGTGATTGGTGGCGCGCTGGCGG-3'
GST-BhuT Forward 2	CAAGCGCGTGATTGTGATTGGTGGCGCGCTGGCGG-3'
GST-BhuT Reverse 1	CTTAGGCCAGGGCATCGCTCAGACGACGGTGC-3'
GST-BhuT Reverse 2	AATTCTTAGGCCAGGGCATCGCTCAGACGACGGTGC-3'
GFP-BhuT Forward 1	GGCCGCAAGCGCGTGATTGTGATTGGTGGCGCGCTGGCGG-3'
GFP-BhuT Forward 2	GCAAGCGCGTGATTGTGATTGGTGGCGCGCTGGCGG-3'
GFP-BhuT Reverse 1	CGGCCAGGGCATCGCTCAGACGACGGTGC-3'
GFP-BhuT Reverse 2	CTAGCGGCCAGGGCATCGCTCAGACGACGGTGC-3'
BhuV E181Q Forward	CTTGACCAACCGACGGCCGCGCTTGACCTTGCG-3'
BhuV E181Q Reverse	CGTCGGTTGGTCAAGCAAAAGGTAGCGAGGACC-3'
BhuU D112V Forward	CGTAATCCACTGGCCGTTCCAGGGCTTGTGGGAGTC-3'
BhuU D112V Reverse	GACTCCCACAAGCCCTGGAACGGCCAGTGGATTACG-3'
BhuU D112A Forward	CGTAATCCACTGGCCGCACCAGGGCTTGTGGGAGTC-3'
BhuU D112A Reverse	GACTCCCACAAGCCCTGGTGCGGCCAGTGGATTACG-3'
BhuU D112R Forward	CGTAATCCACTGGCCCGCCCAGGGCTTGTGGGAGTC-3'
BhuU D112R Reverse	GACTCCCACAAGCCCTGGGCGGGCCAGTGGATTACG-3'

The codons including the mutated nucleotides are underlined.
